# Adenosine closes the K^+^ channel K_Ca_3.1 in human lung mast cells and inhibits their migration *via* the adenosine A_2A_ receptor

**DOI:** 10.1002/eji.200637024

**Published:** 2007-06

**Authors:** S Mark Duffy, Glenn Cruse, Christopher E Brightling, Peter Bradding

**Affiliations:** Department of Infection, Immunity and Inflammation, Institute for Lung Health, University of LeicesterLeicester, UK

**Keywords:** Adenosine, Chemotaxis, Ion channel, K_Ca_3.1, Mast cell

## Abstract

Human lung mast cells (HLMC) express the Ca^2+^-activated K^+^ channel K_Ca_3.1, which opens following IgE-dependent activation. This hyperpolarises the cell membrane and potentiates both Ca^2+^ influx and degranulation. In addition, blockade of K_Ca_3.1 profoundly inhibits HLMC migration to a variety of diverse chemotactic stimuli. K_Ca_3.1 activation is attenuated by the β_2_adrenoceptor through a G_αs_-coupled mechanism independent of cyclic AMP. Adenosine is an important mediator that both attenuates and enhances HLMC mediator release through the G_αs_-coupled A_2A_ and A_2B_ adenosine receptors, respectively. We show that at concentrations that inhibit HLMC degranulation (10^–5^–10^–3^ M), adenosine closes K_Ca_3.1 both dose-dependently and reversibly. K_Ca_3.1 suppression by adenosine was reversed partially by the selective adenosine A_2A_ receptor antagonist ZM241385 but not by the A_2B_ receptor antagonist MRS1754, and the effects of adenosine were mimicked by the selective A_2A_ receptor agonist CGS21680. Adenosine also opened a depolarising current carried by non-selective cations. As predicted from the role of K_Ca_3.1 in HLMC migration, adenosine abolished HLMC chemotaxis to asthmatic airway smooth muscle-conditioned medium. In summary, the G_αs_-coupled adenosine A_2A_ receptor closes K_Ca_3.1, providing a clearly defined mechanism by which adenosine inhibits HLMC migration and degranulation. A_2A_ receptor agonists with channel-modulating function may be useful for the treatment of mast cell-mediated disease.

## Introduction

Mast cells are bone marrow-derived cells that are present in all organs throughout the human body, both at mucosal surfaces and within connective tissues. Mast cells play a major role in tissue homeostasis, host defence and the pathophysiology of many diverse diseases 1. They are best known for their role in asthma and allergy through the ability of allergen to cross-link allergen-specific IgE bound to the high-affinity IgE receptor (FcεRI) expressed on the mast cell surface 2, 3. FcεRI cross-linking triggers a complex signalling cascade, which culminates in the influx of extracellular Ca^2+^ and the release of a plethora of autacoid mediators, proteases and cytokines 1. In addition, the mast cell is activated by many non-immunological stimuli such as cytokines 4, Ig free light chains 5, Toll-like receptor ligands 6–8 and proteases 9, which contribute to the activation of mast cells in response to a variety of tissue insults. In many diseases mast cells re-locate to specific compartments within tissue, such as the airway smooth muscle 10 and submucosal glands 11 in asthma. Drugs that target this migration may prove particularly effective in the treatment of mast cell-mediated disease.

Ion channels are emerging as interesting targets for the modulation of biological function in both inflammatory and structural non-excitable cells 12, 13. Following FcεRI cross-linking, human lung mast cells (HLMC) open the intermediate conductance Ca^2+^-activated K^+^ channel K_Ca_3.1 (also known as IK_Ca_1/K_Ca_4), which hyperpolarises the cell membrane potential to around –45 mV 14. The negative cell membrane potential generated by these open K^+^ channels enhances HLMC Ca^2+^ influx and histamine release 14, 15 due to both the favourable electrical driving force for Ca^2+^ entry and enhanced Ca^2+^ conductance through store-operated Ca^2+^ channels that mediate Ca^2+^ influx following cell activation 16. Also of great interest, blockade of K_Ca_3.1 virtually abolishes HLMC migration to a number of diverse chemotactic stimuli including asthmatic airway smooth muscle-conditioned medium 17.

K_Ca_3.1 in HLMC is closed by the β_2_-adrenoceptor agonist salbutamol 18. This occurs through a G_αs_ G protein-coupled mechanism that is independent of cAMP and explains in part how β_2_-adrenoceptor stimulation translates into reduced secretion. Whether this is specific to the β_2_-adrenoceptor or whether K_Ca_3.1 is modulated by other G protein-coupled receptors (GPCR) is therefore of great interest. Adenosine is a purine nucleoside generated by numerous cell types in response to cell stress and hypoxia. It modulates human mast cell secretion through the adenosine A_2A_ and A_2B_ GPCR and is of particular relevance to asthma 19–21. The A_2A_ receptor signals *via* adenylate cyclase, involving G_αs_ coupling, and A_2B_ signals *via* multiple mechanisms including adenylate cyclase, diacylglycerol and inositol triphosphate, involving both G_αs_ and G_αq_ coupling 22. A_2B_ signalling has additionally been show to be mediated independently of G proteins *via* PDZ-containing proteins. The effects of adenosine on HLMC both *in vitro* and *in vivo* are complex; at relatively low concentrations *in vitro* (10^–6^ M), adenosine is said to potentiate IgE-dependent secretion, but the reported effects vary widely 23–27. Because adenosine induces cytokine secretion from a human mast cell line through the A_2B_ receptor 28, it is also thought that potentiation of secretion from HLMC is mediated *via* the A_2B_ receptor, but this is not proven. In contrast, at higher concentrations of adenosine (10^–5^ to 10^–3^ M), there is more consistent dose-dependent and profound inhibition of secretion 23, 24, 27, 29, mediated predominantly *via* the A_2A_ receptor 30. *In vivo*, adenosine induces histamine and tryptase release from resident mast cells when delivered as an aerosol to the airways 31 and induces bronchoconstriction in subjects with asthma but not normal subjects. It is thought that these *in vivo* effects are mediated directly on mast cells, although it remains possible that intermediaries and neural reflexes are also involved 32.

Since K_Ca_3.1 in HLMC is closed by the G_αs_-coupled β_2_-adrenoceptor, we hypothesised that activation of the A_2A_ G_αs_-coupled adenosine receptor would also close K_Ca_3.1 in these cells. Furthermore, if adenosine was to close K_Ca_3.1, then it should inhibit HLMC chemotaxis. To test this hypothesis, we used the patch-clamp technique to investigate the effects of adenosine on HLMC ion channel function and investigated the effect of adenosine on HLMC migration in response to asthmatic airway smooth muscle-conditioned medium.

## Results

### Inhibition of HLMC IgE-dependent histamine release by adenosine

**Figure 1:**
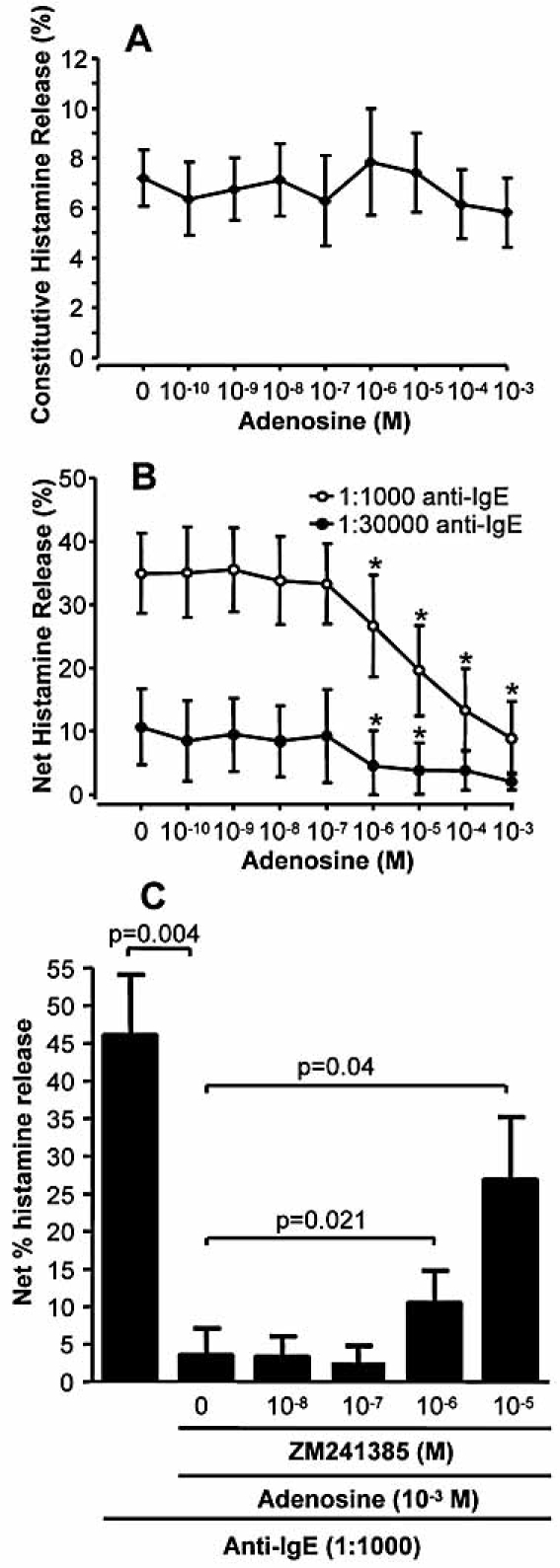
Effects of adenosine on histamine release from HLMC. (A) Effect of adenosine on constitutive histamine release from HLMC. The mean ± SEM of four experiments is shown. (B) Effect of adenosine on IgE-dependent histamine release from HLMC following a maximal (1:1000 anti-IgE) or submaximal (1:30 000 anti-IgE) activation stimulus. The mean ± SEM of four experiments is shown (*p<0.05 compared to control). (C) Inhibition of histamine release by adenosine is attenuated by the adenosine A_2A_ receptor antagonist ZM241385. The mean ± SEM of four experiments is shown. Donors are different from those in (A) and (B).

Adenosine alone had no effect on mast cell histamine release in the dose range 10^–10^ to 10^–3^ M (Fig. 1A). In the presence of anti-IgE, no potentiation of IgE-dependent histamine release by adenosine was evident with either maximal (1:1000 anti-IgE) or sub-maximal (1:30 000 anti-IgE) activation (Fig. 1B). However, there was dose-dependent inhibition of IgE-dependent histamine release over the dose range 10^–6^ to 10^–3^ M adenosine (Fig. 1B). Half-maximal suppression (IC_50_) of histamine release occurred at an adenosine concentration of 73.8±23.5 μM. This inhibition of histamine release by adenosine was significantly attenuated in the presence of the adenosine A_2A_ receptor antagonist ZM241385 (Fig. 1C).

### Adenosine alone does not open K_Ca_3.1 but opens an outwardly rectifying non-selective cation current

**Figure 2:**
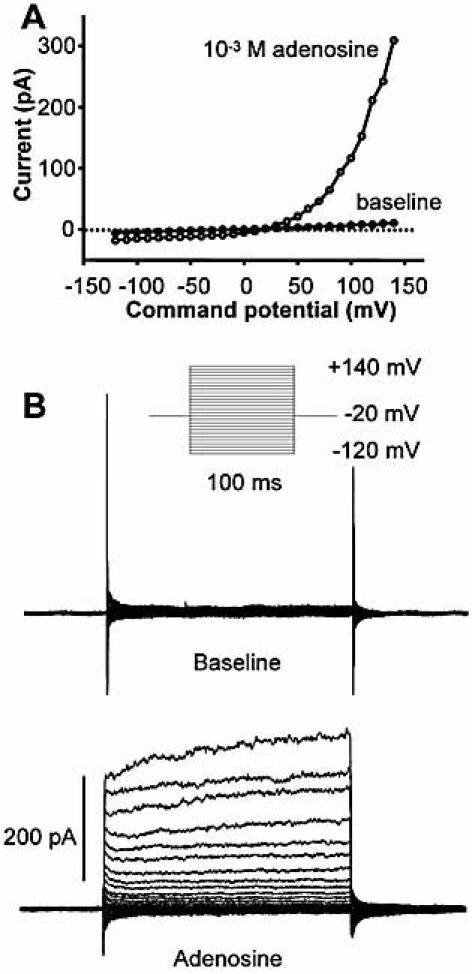
Adenosine activates an outwardly rectifying non-selective cation current in HLMC. (A) Current-voltage curve of the outwardly rectifying current induced by 10^–3^ M adenosine in a resting HLMC. (B) Raw current from the same cell as in (A) demonstrating instantaneous activation following voltage steps and slight accentuation during a 100 millisecond pulse. The voltage protocol is shown inset.

Adenosine at around 10^–6^ M has been reported to enhance IgE-dependent histamine release, although this was not the case in our hands. We therefore first examined the effects of adenosine alone in the concentration range 10^–7^ to 10^–3^ M to assess whether it might open K_Ca_3.1, but it did not. However, adenosine at 10^–4^ and 10^–3^ M did open a novel strongly outwardly rectifying current (Fig. 2A). With an adenosine concentration of 10^–3^ M, this current appeared in 15/18 cells tested (*n*=6 donors) within 30 s of adding adenosine to the recording chamber, consistently reached maximal amplitude over the course of a few seconds, and typically “ran-down” completely over 20 s. This sequence could be re-initiated on further application of adenosine, but the transient nature of the current prevented detailed electrophysiological characterisation. In cells in which the outward current appeared, adenosine at a concentration of 10^–3^ M increased membrane current measured at +100 mV from 13.0±3.4 pA to 153.0±27.0 pA (*n*=15), with a positive shift in reversal potential from –14.4±3.4 mV to +5.9±9.8 mV. This current demonstrated instantaneous activation following voltage steps and increased slightly in amplitude over the course of a 100-millisecond pulse (Fig. 2B). There was no significant change in reversal potential or mean current at +100 mV when recording with extracellular Na^+^ methanesulphonate (162.2±62.1 pA, *n*=4, *p*=0.89) or N-methyl-D-glucamine (NMDG) Cl^–^ (180±48.2 pA, *n*=7, *p*=0.61) rather than NaCl, and there was no change when recording with intracellular Cs^2+^ glutamate (142.0±36.3 pA, *n*=4, *p*=0.928) rather than KCl. However, replacing intracellular K^+^ with N-methyl-D-glucamine markedly reduced the outward current at +100 mV (31.7±6.0 pA, *n*=6, *p*=0.0013) but did not alter the reversal potential (Vm 5.8±3.5 mV, *p*=0.99). The ions carried by this current are therefore non-selective cations. Lastly, the selective adenosine A_2A_ receptor agonist CGS21680 did not induce this outward current, and the current was still inducible in the presence of the selective A_2A_ and A_2B_ receptor antagonists ZM241385 33 (120.6±52.6 pA at +100 mV, *n*=5, *p*=0.56) and MRS1754 34 (160.2±66.9 pA at +100 mV, *n*=5, *p*=0.91), respectively. Taken together this suggests that induction of this non-selective cation current does not involve the A_2A_ or A_2B_ adenosine receptors.

### Adenosine closes K_Ca_3.1 in the presence of the specific K_Ca_3.1 opener 1-EBIO

We next examined whether adenosine closes K_Ca_3.1 at concentrations that inhibit IgE-dependent degranulation. Because adenosine might potentially inhibit many cell activation pathways that could reduce cytosolic free Ca^2+^ and thus reduce K_Ca_3.1 activity indirectly, we concentrated on studying the effects of adenosine on K_Ca_3.1 currents that were induced by the K_Ca_3.1 opener 1-ethyl-2-benzimidazolinone (1-EBIO). This compound opens K_Ca_3.1 with a half-maximal value of about 30 μM for heterologously expressed K_Ca_3.1, with a maximal effect at about 300 μM 35. The effects of 1-EBIO are relatively specific for K_Ca_3.1, opening it by enhancing its sensitivity to [Ca^2+^]_i_ 35. Thus at 100 μM 1-EBIO, maximal K^+^ currents are achieved in the presence of 100 nM free Ca^2+^,which is below the resting [Ca^2+^]_i_ of most cell types, including HLMC 15.

**Figure 3:**
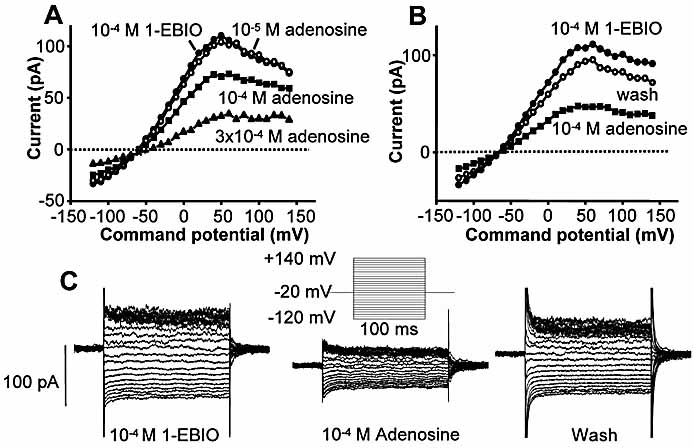
Adenosine closes K_Ca_3.1 in HLMC. Current-voltage curves demonstrating (A) dose-dependent suppression of 1-EBIO-induced K_Ca_3.1 current by adenosine and (B) reversibility of adenosine-mediated suppression of K_Ca_3.1 following removal of adenosine (wash). (C) Raw currents from the same cell as illustrated in (B) above.

**Figure 4:**
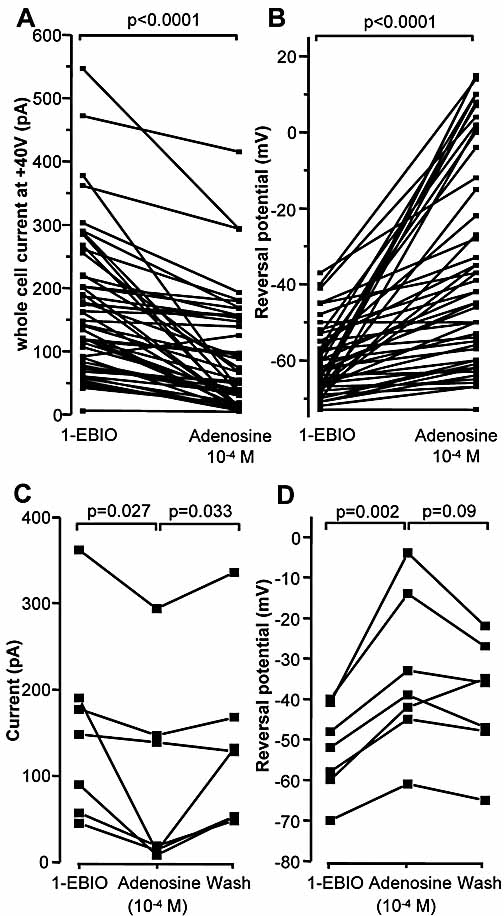
Suppression of K_Ca_3.1 by adenosine is consistent and reversible. (A) Suppression of K_Ca_3.1 current measured at +40 mV by 10^–4^ M adenosine (*n*=49 cells). (B) Shift in whole-cell current reversal potential (Vm) by 10^–4^ M adenosine (*n*=49 cells). (C) K_Ca_3.1 current measured at +40 mV after addition of 1-EBIO, suppression by 10^–4^ M adenosine and then reversibility of suppression following removal of adenosine (wash) (*n*=7 cells). (D) Whole-cell current reversal potential (Vm) after the addition of 1-EBIO, a depolarising positive shift in response to 10^–4^ M adenosine and then reversibility following removal of adenosine (wash) (*n*=7 cells).

At concentrations of 10^–6^ M and below, adenosine had little or no effect on K_Ca_3.1 currents that had been activated by 1-EBIO. However, addition of adenosine (10^–5^ to 10^–3^ M) to cells in which K_Ca_3.1 had been activated by 1-EBIO produced a rapid dose-responsive inhibition of channel activity with an associated positive shift in membrane potential (Fig. 3A). Adenosine at 10^–4^ M suppressed the K_Ca_3.1 current in >90% of cells (Fig. 4A). Thus addition of 10^–4^ M adenosine reduced K_Ca_3.1 membrane current at +40 mV from 169.3±16.1 to 84.8±12.6 pA (*n*=49 cells, *p*<0.0001)(Fig. 4A), with a corresponding shift in reversal potential (Vm) from −61.0±1.3 to −35.1±4.0 mV (*p*<0.0001) (Fig. 4B). The IC_50_ of K_Ca_3.1 by adenosine occurred at 30.9±12.7 μM (calculated from 5 cells). Importantly, the effect of adenosine was partially reversed within 1 min by removing it from the recording solution (current post-adenosine 90±41 pA, post-wash 131±39 pA, *n*=7, *p*=0.035; Vm post-adenosine −34.0±7.3 mV, post-wash −40±5.5 mV, *p*=0.095) (Figure 3B, C and 4C, D), indicating that non-specific “run-down” was not responsible for the effects seen. Because the outwardly rectifying current described above rapidly faded, this did not interfere with analysis of the K_Ca_3.1 current.

### K_Ca_3.1 modulation by adenosine is mediated *via* A_2A_ but not A_2B_ receptors

**Figure 5:**
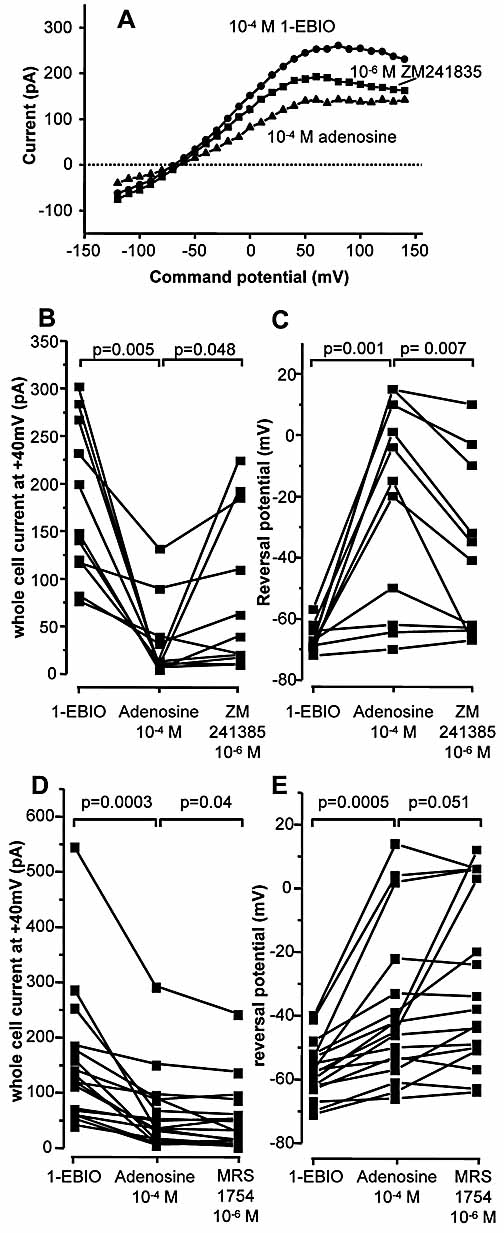
Effects of adenosine receptor antagonists on adenosine-dependent closure of K_Ca_3.1. (A) Current-voltage curve demonstrating the reversibility of K_Ca_3.1 suppression of adenosine by the A_2A_ receptor antagonist ZM241385. (B) K_Ca_3.1 current measured at +40 mV after addition of 1-EBIO, suppression by 10^–4^ M adenosine and then reversibility of suppression following addition of the A_2A_ receptor antagonist ZM241385 (*n*=11 cells). (C) Whole-cell current reversal potential (Vm) after the addition of 1-EBIO, a depolarising positive shift in response to 10^–4^ M adenosine, and then reversibility following addition of the A_2A_ receptor antagonist ZM241385 (*n*=11 cells). (D) K_Ca_3.1 current measured at +40 mV after addition of 1-EBIO, suppression by 10^–4^ M adenosine and then failure to reverse following addition of the A_2B_ receptor antagonist MRS1754 (*n*=17 cells). (E) Whole-cell current reversal potential (Vm) after the addition of 1-EBIO, a depolarising positive shift in response to adenosine and then failure to reverse following addition of the A_2B_ receptor antagonist MRS1754 (*n*=17 cells).

To confirm that the effects of adenosine were mediated *via* adenosine receptors, we examined the effects of A_2A_ and A_2B_ receptors agonists/antagonists. The suppression of K_Ca_3.1 by adenosine was partially reversed by the competitive A_2A_ receptor antagonist ZM241385 (Fig. 5A–C). Thus in experiments studying ZM241385 at a concentration of 10^–6^ M, current at +40 mV was 32.5±12.2 pA post-adenosine, increasing to 83.4±24.4 pA post-ZM241385 (*p*=0.048, *n*=11 cells)(Fig. 5B). There were parallel shifts in reversal potential (Vm post-adenosine −22.3±10.1 mV, post-ZM241385 −39.5±8.4 mV, *p*=0.007) (Fig. 5C). However, unlike the β_2_-adrenoceptor inverse agonist ICI 118551 18, ZM241385 did not open K_Ca_3.1 on its own (data not shown, *n*=10 cells tested). The effects of adenosine on the K_Ca_3.1 current were not mediated *via* the A_2B_ receptor, as the selective A_2B_ receptor antagonist MRS1754 (up to 10^–6^ M) did not reverse the suppressive effects of adenosine (in fact there was a further small but significant decline in current amplitude) (Fig. 5D, E). When comparing the effects of ZM241385 with MRS1754 regarding their ability to reverse the adenosine-suppressed K_Ca_3.1 current, there was a highly significant difference (*p*=0.008).

**Figure 6:**
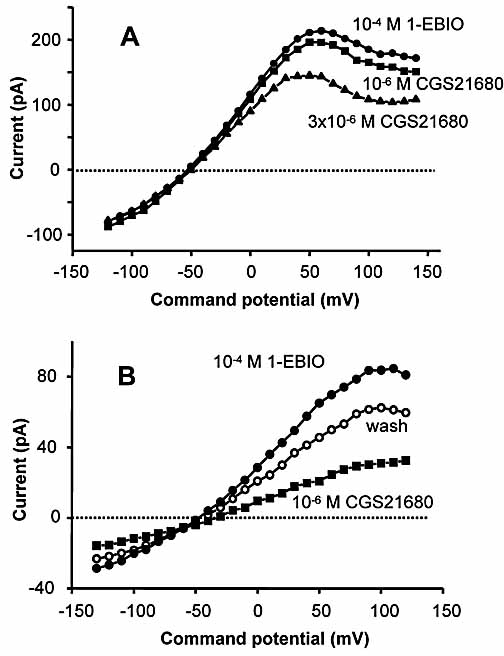
The effect of an adenosine A_2A_ receptor agonist on K_Ca_3.1 currents in HLMC. (A) Current-voltage curve demonstrating dose-dependent suppression of a 1-EBIO-induced K_Ca_3.1 current by the A_2A_ receptor agonist CGS21680 and (B) reversibility of this suppression following its removal (wash).

In further support of the A_2A_ adenosine receptor coupling to the K_Ca_3.1 channel, the selective A_2A_ receptor agonist CGS21680 mimicked the effects of native adenosine in a dose-dependent and partially reversible manner (Fig. 6). At a concentration of 10^–6^ M, CGS21680 reduced the K_Ca_3.1 current from 118.8±24.1 pA to 97.4±21.8 pA (*p*=0.007, *n*=9).

**Figure 7:**
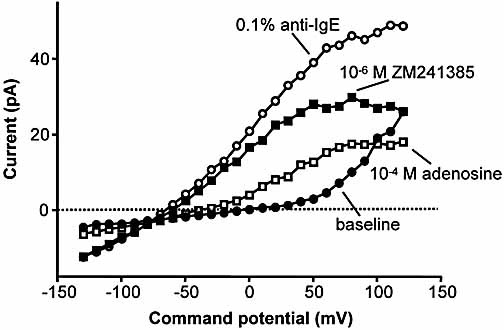
The effect of adenosine on K_Ca_3.1 currents elicited by anti-IgE-dependent mast cell activation. Data from a representative HLMC showing K_Ca_3.1 current opening following anti-IgE-dependent activation, suppression of this by adenosine and reversal of the adenosine-induced suppression by the adenosine A_2A_ receptor antagonist ZM241385.

Lastly, we confirmed that the adenosine-dependent regulation of K_Ca_3.1 was relevant to K_Ca_3.1 channels that had been opened by anti-IgE-dependent activation. Anti-IgE (1:1000 dilution) opened K_Ca_3.1 in 6/6 cells tested (baseline current at +40 mV 17.5±7.3 pA, baseline Vm −34.5±8.0 mV; post anti-IgE current 81.5±18.0 pA, Vm −62.3±2.5 mV). Adenosine (10^–4^ M) suppressed the current to 49.3±12.6 pA post-adenosine (*p*=0.007) and produced an associated positive shift in Vm (−49.2±6.5 mV, *p*=0.046). This suppressive effect of adenosine was partially reversed by ZM241385 (current 75.6±19.8 pA, *p*=0.050; Vm −58.5±4.8 mV, *p*=0.020)(Fig. 7).

### Adenosine inhibits HLMC migration

**Figure 8:**
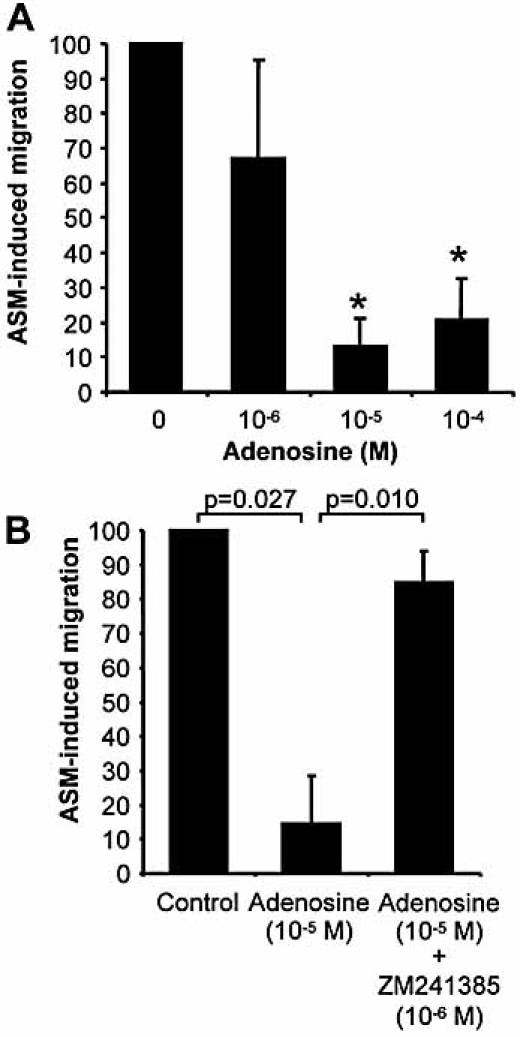
Inhibition of HLMC chemotaxis by adenosine. (A) Conditioned medium from asthmatic airway smooth muscle (ASM) was used as the chemotactic stimulus. Data represents the mean ± SEM from five individual HLMC donors (*p<0.005). (B) Inhibition of HLMC migration by adenosine is prevented by the A2A receptor antagonist ZM241385. Data represents the mean ± SEM from three individual HLMC donors.

Conditioned medium from asthmatic airway smooth muscle that has been activated with TNF-α, IFN-γ and IL-1β mediates HLMC chemotaxis predominantly *via* the CXCL10/CXCR3 pathway, with additional contributions from ligands for CXCR1 and CXCR3 36. Inhibition of K_Ca_3.1 by channel blockers markedly suppresses this HLMC chemotaxis 17. Migration of HLMC using conditioned medium from asthmatic airway smooth muscle was 2.0±0.7-fold higher than in the presence of control medium (*n*=5, *p*=0.029), and this was almost completely abrogated by adenosine, with a calculated IC_50_ of 2.5±0.8 μM (Fig. 8A). This inhibitory effect of adenosine on HLMC migration utilised the A_2A_ adenosine receptor, as it was blocked by the A_2A_ receptor antagonist ZM241385 (10^–6^ M) (*n*=3, *p*=0.010) (Fig. 8B).

## Discussion

In this study we have tested the novel hypothesis that modulation of HLMC activation by adenosine interferes with ion channel function in these cells. We show for the first time that adenosine closes the K_Ca_3.1 K^+^ channel *via* the G_αs_-coupled A_2A_ adenosine receptor and that, as predicted from this effect, adenosine markedly attenuates HLMC migration. Since opening of the K_Ca_3.1 K^+^ channel potentiates Ca^2+^ influx and histamine secretion 14 and its blockade almost abolishes chemotactic responses 17, the ability of adenosine to close this channel provides a potential mechanism directly linking A_2A_ receptor engagement with diminished secretion and migration.

Adenosine closed K_Ca_3.1 following IgE-dependent activation, but it was possible that this effect might occur indirectly through the perturbation of complex intracellular signalling pathways. We therefore used the K_Ca_3.1 opener 1-EBIO to activate the channel rather than IgE-dependent activation. Since 1-EBIO opens K_Ca_3.1 in resting cells by increasing its affinity for Ca^2+^ 35, it is particularly interesting that adenosine closes K_Ca_3.1 under these conditions. This effect of adenosine was reversed both by removing it from the recording solution and by the addition of the competitive A_2A_ adenosine receptor antagonist ZM241385, indicating a receptor-mediated mechanism. Since the effects of adenosine were not antagonised by the A_2B_ receptor antagonist MRS1754 but were mimicked by the A_2A_ receptor agonist CGS21680, we have firm evidence that the suppression of K_Ca_3.1 by adenosine is mediated *via* the A_2A_ adenosine receptor.

The ability of adenosine to close K_Ca_3.1 is in keeping with our previous observations that this channel is closed by the G_αs_-coupled β_2_-adrenoceptor 18. These effects of both A_2A_ adenosine and β_2_-adrenoceptor activation on K_Ca_3.1 are not mimicked by cAMP analogues or the activator of adenylate cyclase forskolin 18, indicating that the most likely mechanism is membrane-delimited involving the G_αs_ or βγ subunits of these GPCR. This is further suggested by the fact that adenosine (and β_2_-receptor agonists) modulate K_Ca_3.1 in the whole-cell configuration of patch-clamp recording. This mode of recording dialyses the cell and depletes soluble intracellular second messengers, indicating that the adenosine A_2A_ receptor and K_Ca_3.1 channel are likely to be coupled tightly in a membrane-restricted signalplex.

Several molecules that attenuate HLMC secretion, including adenosine and β_2_-adrenoceptor agonists, increase intracellular cAMP. It is this increase in intracellular cAMP that has been thought to couple to inhibition of secretion, but there is no mechanism to explain this. However, the exclusive role of cAMP in the inhibition of mast cell degranulation and promotion of smooth muscle relaxation has recently been challenged 37, 38. We have shown previously that opening of K_Ca_3.1 enhances IgE-dependent Ca^2+^ influx and degranulation 15 and that its blockade attenuates this 14. Thus the demonstration that adenosine closes K_Ca_3.1 provides for the first time a clearly defined mechanism by which adenosine A_2A_ receptor stimulation can be linked to attenuated secretion.

The migration and re-location of mast cells within tissues is important for their effector function in a number of diseases 10, 39, 40. For example in asthma, the role of mast cells in the disordered airway physiology is facilitated by their migration into the airway epithelium 41, submucosal glands 11 and airway smooth muscle 10. Inhibition of their migration and subsequent microlocalisation within these structures is therefore an attractive therapeutic target. Blockade of K_Ca_3.1 markedly inhibits HLMC migration in response to a number of diverse chemotactic stimuli, including conditioned medium from activated asthmatic airway smooth muscle 17. The ability of adenosine to close K_Ca_3.1 suggested it should also inhibit HLMC migration, which was indeed the case. Adenosine was slightly more potent at inhibiting migration (IC_50_ ∼3 μM) than closing K_Ca_3.1 (IC_50_ ∼31 μM) or inhibiting mediator release (IC_50_ ∼74 μM). These results regarding migration *versus* degranulation are consistent with the observation that HLMC migration is also far more sensitive to direct K_Ca_3.1 blockade than degranulation 14, 17. Migration appears particularly sensitive to K_Ca_3.1 blockade, because even a partial block markedly inhibits oscillations in K_Ca_ activity, which in turn prevents the changes in cell volume that are required for migration to proceed 42, 43. This is further supported by the observation that in addition to blocking of K_Ca_3.1, permanently opening it with 1-EBIO also inhibits migration 44. However, the relative sensitivity of migration to adenosine *versus* K_Ca_3.1 closure might also suggest that the effects of adenosine on migration operate through additional mechanisms, such as generation of cAMP. However, the experimental conditions are also likely to account for some of this difference. This is because we can only reliably patch-clamp HLMC at temperatures up to 27°C 14, which will likely slow down the kinetics and magnitude of any agonist effect, and we have also recorded currents in the whole-cell configuration, which dialyses the cell, resulting in the loss of potentially important signalling components. In contrast, cell migration and mediator release assays are performed in intact cells at 37°C.

Previous studies have suggested that at lower concentrations (approximately 10^–6^ M), adenosine actually promotes mediator release from human mast cells 23–27. However, the magnitude of this effect and the concentrations of adenosine required to produce it have been highly varied 23–27, 29, and a recent study investigating cord blood-derived human mast cells did not find any potentiating effect 30. Adenosine did not potentiate degranulation from HLMC in our hands, which is in keeping with a failure to detect any modulation of ion channel activity, such as opening of K_Ca_3.1 either directly or indirectly through initiation of Ca^2+^ influx, which might be predicted to enhance secretion. Interestingly, in rodent mast cells, adenosine opens an outwardly rectifying K^+^ channel that is not Ca^2+^-activated, is distinct from K_Ca_3.1 and has been proposed to account for the ability of adenosine to enhance mediator release in these cells 45, 46. We did not see this channel in HLMC, which is yet another example of the important heterogeneity evident in the biology of human and rodent mast cells 47.

In addition to its effects on K_Ca_3.1, adenosine at relatively high concentrations opened a novel and transient outwardly rectifying ion channel that was indirectly demonstrated to carry non-selective cations. Interestingly, this current still appeared in the presence of the adenosine A_2A_ and A_2B_ receptor antagonists, suggesting that it is mediated through another pathway. Because this outwardly rectifying current depolarises the cells (and would therefore be predicted to attenuate Ca^2+^ influx) and occurs at concentrations of adenosine that attenuate degranulation, it is possible that this channel also inhibits mast cell mediator release. The transient nature of this current might be due to “run-down” caused by the depletion of intracellular second messengers induced by whole-cell recording, but we believe that this is unlikely due to the very rapid nature by which the current diminishes. However, it will be important that this is analysed in more detail using the perforated patch mode of recording.

*In vivo*, adenosine induces airway narrowing in asthmatic subjects, with evidence that this results from mast cell degranulation 20, 21. This effect is thought to be mediated directly *via* the A_2B_ adenosine receptor on mast cells, although this is not proven, and might also involve indirect mechanisms 28, 32. Adenosine A_2B_ receptor antagonists are therefore being considered as novel therapies for asthma. An alternative strategy is to target the anti-inflammatory A_2A_ receptor with specific agonists delivered locally to the airway 48, 49. Drugs that block K_Ca_3.1 are also in development as anti-inflammatory treatments 13. Our demonstration that the A_2A_ adenosine receptor closes K_Ca_3.1 in HLMC therefore provides further support for the development of specific A_2A_ adenosine receptor agonists for the treatment of mast cell-mediated disease.

## Materials and methods

### Reagents

We used the following reagents: stem cell factor (SCF), IL-6 and IL-10 (R&D, Abingdon, UK); goat polyclonal anti-human IgE, adenosine, CGS21680, ZM241385, MRS1754 (Sigma, Poole, Dorset, UK); 1-ethyl-2-benzimidazolinone (1-EBIO) (Tocris, Avonmouth, UK); human myeloma IgE (Calbiochem-Novabiochem, Nottingham, UK); mouse IgG_1_ mAb YB5B8 (anti-CD117) (Cambridge Bioscience, Cambridge, UK); sheep anti-mouse IgG_1_ Dynabeads (Dynal, Wirral, UK); Dulbecco's Modified Essential Medium (DMEM)/glutamax/Hepes, antibiotic/antimycotic solution, MEM nonessential amino acids and FCS (Life Technologies, Paisley, Scotland, UK).

### Human mast cell purification and culture

All human subjects gave written informed consent, and the study was approved by the Leicestershire Research Ethics Committee. HLMC were dispersed and purified from macroscopically normal lung (*n*=11 donors) obtained within 1 h of resection for lung cancer using immunomagnetic affinity selection as described previously 50. Final mast cell purity was >99%, and viability was >97%. HLMC were cultured in DMEM/glutamax/Hepes containing antibiotic/antimycotic solution, nonessential amino acids, 10% FCS, 100 ng/mL SCF, 50 ng/mL IL-6 and 10 ng/mL IL-10 for up to 10 wks as described previously 15, 51.

### HLMC activation

For analysis of histamine release, 1 × 10^4^ mast cells were warmed to 37°C in triplicate in 50 μL DMEM. Adenosine (± ZM241385 or DMSO control) at 4× the final concentration in 25 μL DMEM was added just prior to addition of 25 μL DMEM containing 4× the final concentration of goat polyclonal anti-human IgE (1:1000 final dilution gives maximal histamine release, 1:30 000 final dilution gives sub-maximal histamine release) 15. After 30 min incubation at 37°C, the cells were centrifuged at 250 × g for 4 min, the supernatant decanted and control cell pellets lysed in sterile deionised water for measurement of total histamine content as described previously 50. The final DMSO concentration was 0.1%.

### Electrophysiology

The whole-cell variant of the patch-clamp technique was used 14, 52. Patch pipettes were made from borosilicate fiber-containing glass (Clark Electromedical Instruments, Reading, UK), and their tips were heat-polished, typically resulting in resistances of 4–6 MΩ. The standard pipette solution contained 140 mM KCl, 2 mM MgCl_2_, 10 mM Hepes, 2 mM Na^+^-ATP and 0.1 mM GTP, pH 7.3. The standard external solution contained 140 mM NaCl, 5 mM KCl, 2 mM CaCl_2_, 1 mM MgCl_2_ and 10 mM Hepes, pH 7.3. For recording, mast cells were placed in 35-mm dishes containing standard external solution. Whole-cell currents were recorded using an Axoclamp 200 A amplifier (Axon Instruments, Foster City, CA, USA), and currents were evoked by applying voltage commands to a range of potentials in 10 mV steps from a holding potential of –20 mV. The currents were digitized (sampled at a frequency of 10 kHz), stored on computer and subsequently analyzed using pClamp software (Axon Instruments). Capacitance transients were minimized using the capacitance neutralization circuits on the amplifier. Correction for series resistance was not routinely applied. Experiments were performed at 27°C, with the temperature controlled by a Peltier device. Experiments were performed with a perfusion system (Automate Scientific, San Francisco, CA) to allow solution changes, although drugs were added directly to the recording chamber.

### HLMC chemotaxis

HLMC chemotaxis assays were performed using the Transwell system (BD Biosciences, Oxford, UK) with 24-well plates as described previously 17, 36. Conditioned medium from asthmatic airway smooth muscle that had been activated with TNF-α, IL-1β and IFN-γ was placed in the lower wells as described previously 36, with appropriate cytokine-containing medium in the negative control. Adenosine was added to the bottom wells in the concentration range 10^–6^–10^–3^ M, and 1 × 10^5^ HLMC in 100 μL were added to the top well. After incubating the cells for 3 h at 37°C, we counted the number of HLMC in the bottom well using Kimura stain in a haemocytometer. HLMC migration was calculated as the fold increase of migrated cells in the test wells compared to the negative control (no chemoattractant in the lower well) as described previously 17, 36.

### Data presentation and statistical analysis

Data are expressed as mean ± SEM unless otherwise stated. Differences between groups of data were explored using Student's paired or unpaired *t-*test (two-tailed) as appropriate.

## Abbreviations:

1-EBIO: 1-ethyl-2-benzimidazolinone
GPCR: G protein-coupled receptor
HLMC: human lung mast cell
